# A versatile approach to evaluate the occurrence of microfibers in mussels *Mytilus galloprovincialis*

**DOI:** 10.1038/s41598-022-25631-2

**Published:** 2022-12-17

**Authors:** Michela Volgare, Serena Santonicola, Mariacristina Cocca, Roberto Avolio, Rachele Castaldo, Maria Emanuela Errico, Gennaro Gentile, Gennaro Raimo, Maurizio Gasperi, Giampaolo Colavita

**Affiliations:** 1 Institute of Polymers, Composites and Biomaterials, National Research Council of Italy, Via Campi Flegrei 34, 80078 Pozzuoli, NA Italy; 2 Department of Medicine and Health Sciences “V. Tiberio”, University of Molise, Via F. De Santis, 86100 Campobasso, Italy

**Keywords:** Environmental sciences, Materials science

## Abstract

Microplastics of fibrous shape are esteemed to be the most abundant micro-debris form present in the environment. Despite the occurrence of microfibers in fish may pose a risk to human health, the literature is scarce regarding studies on the contamination in commercial marine fish mostly due to methodological issues. In this study, a versatile approach, able to discriminate among natural and synthetic microfibers according to the evaluation of specific morphological features, is proposed in farmed mussels (*Mytilus galloprovincialis*). The approach was useful to determine that microfibers were present in 74% of mussel samples, with a mean number of 14.57 microfibers/individual, corresponding to 3.13 microfibers/g w.w. A negative correlation between the size of analysed mussels and the amount of microfibers/g w.w. was detected, showing that smaller specimens contained more microfibers than the larger ones. This work paves the way to further studies aimed to adequately assess the risk that microfibers may pose to marine biota, also considering the commercial value as seafood items of many species of the *Mytilus* genus and the potential implication for human exposure.

## Introduction

The marine ecosystem is the final endpoint of plastic waste that can affect a variety of organisms, including fish and invertebrates^1^. Microplastics (< 5 mm) have been found worldwide in the digestive tracts of marine and freshwater animals, including commercially important species, with the highest concentrations in animals at the base of the marine food web, like bivalve shellfish^2–7^. The exposure by organisms may lead to several adverse effects, like blockage of the intestinal tract, reduction of feeding stimuli, and lack of reproduction^8^. Microplastics can also concentrate organic pollutants from surrounding water, which can then be released into the organisms upon ingestion^9^.

Recently, new emerging debris has been considered, represented by synthetic and natural microfibers (below 5 mm in length), coming from the textile industry or urban wastewaters^10–12^. Synthetic microfibers represent the major microplastic form found in environmental samples, and in the gut of diverse marine species^13–17^. However, recent studies have also revealed the occurrence of a considerable number of natural microfibers (cotton) and artificial microfibers (rayon)^10,18,19^. Similar to the other type of microplastic, microfibers of natural origin seem to have both physical and toxicological deleterious effects. Natural microfibers may be harmful as synthetic microfibers, especially as a carrier of associated chemicals such as dyes and additives^10^. However, there has been little information to date on the interactions between marine fauna and microfibers, mostly because in some studies microfibers were excluded due to methodological issues, as high risk of airborne contamination during sampling and processing^16,19,20^. Nevertheless, microfibers may present a small diameter compatible with the feeding size range (around 15–30 μm) of filter feeding organisms, which allows their entrance in the bivalve digestive system through the narrower width, avoiding the mechanisms of the bivalves to filter out particles larger than ~ 100 μm^21,22^.

Mussels are interesting species to evaluate risks associated with plastic debris in marine habitats because, as benthic filter feeding organisms, they may accumulate microplastics and contribute to their transfer towards the marine trophic web^23,24^. Microplastics have been shown to accumulate also in the mussel gills and to adhere to organs not associated with feeding, including foot and mantle^8^. Synthetic microfibers, in particular, are among the most frequently microplastics found in mussels^9,16,25^. These particles were detected in mussels from Scotland^14^, Swedish rivers^26^, Italy^13^, and California^16,27^. The geometry of microfibers may allow them to be trapped into gills and hepatopancreas and cannot be easily removed by mussels accumulating into them^24^.

The Mediterranean Sea offers a privileged space to assess plastic contamination in mussels, being one of the most affected areas in the world^28^, probably due to the morphological characteristics of the basin, the high human population density, and the intense anthropogenic activities^28,29^. Plastic pollution appears to be ubiquitous in the Tyrrhenian Sea, the western side of the Mediterranean basin^30,31^, where a high number of microfibers were found in the gut of commercially important species collected in this area, such as *Zeus faber, Lepidopus caudatus*^32^*,* *Pagellus* spp^33^*,* *Sardina pilchardus, Engraulis encrasicolus*^34^*, Mullus barbatus, Trigla lyra, Galeus melastomus, Scyliorhinus canicula* and *Raja miraletus*^35^. However, little information on microfiber contamination in mussels coming from the Tyrrhenian Sea is available^24^.

This study aims to propose a versatile approach to evaluate the occurrence of natural and synthetic microfibers in farmed mussels (*Mytilus galloprovincialis*) from the Tyrrhenian Sea (Western Mediterranean Sea) sold for human consumption.

The purified samples are usually examined by different analytical techniques, among which the most commonly used are Fourier-transform infrared spectroscopy (FTIR), and Raman spectroscopy, which identified microplastics through their vibrational spectrum. However, the spectroscopic approach presents drawbacks that are mainly correlated with the choice of appropriate filters, flat samples, appearance of fluorescence phenomena and the analysis may be time consuming and labor intensive^36^.

In this respect, an optical method was applied and implemented, using morphological features of microfibers, for the identification and quantification of microfibers in mussels. Morphological analysis of the microfiber represents a fundamental tool for the identification of the family of textile fibres. In fact, the analysis of microfibers under an optical or electronic microscope allows the identification of the typical morphological features of the fibers on the basis of which it is possible to identify their origin^37,38^.

The results allow the indication of the applicability of the proposed approach to monitor the microfiber occurrence in marine organisms.

## Results

### Abundance of microfibers in mussels

Overall, 50 farmed mussels from the Tyrrhenian Sea sold for human consumption were analyzed. The mean bivalves’ length and weight (± SD) were 6.43 ± 1.02 cm and 5.89 ± 2.47 g. Microfibers were the predominant particle form detected on the filters. In total, 1,401 microfibers were counted in the analyzed samples.

**Figure 1:**
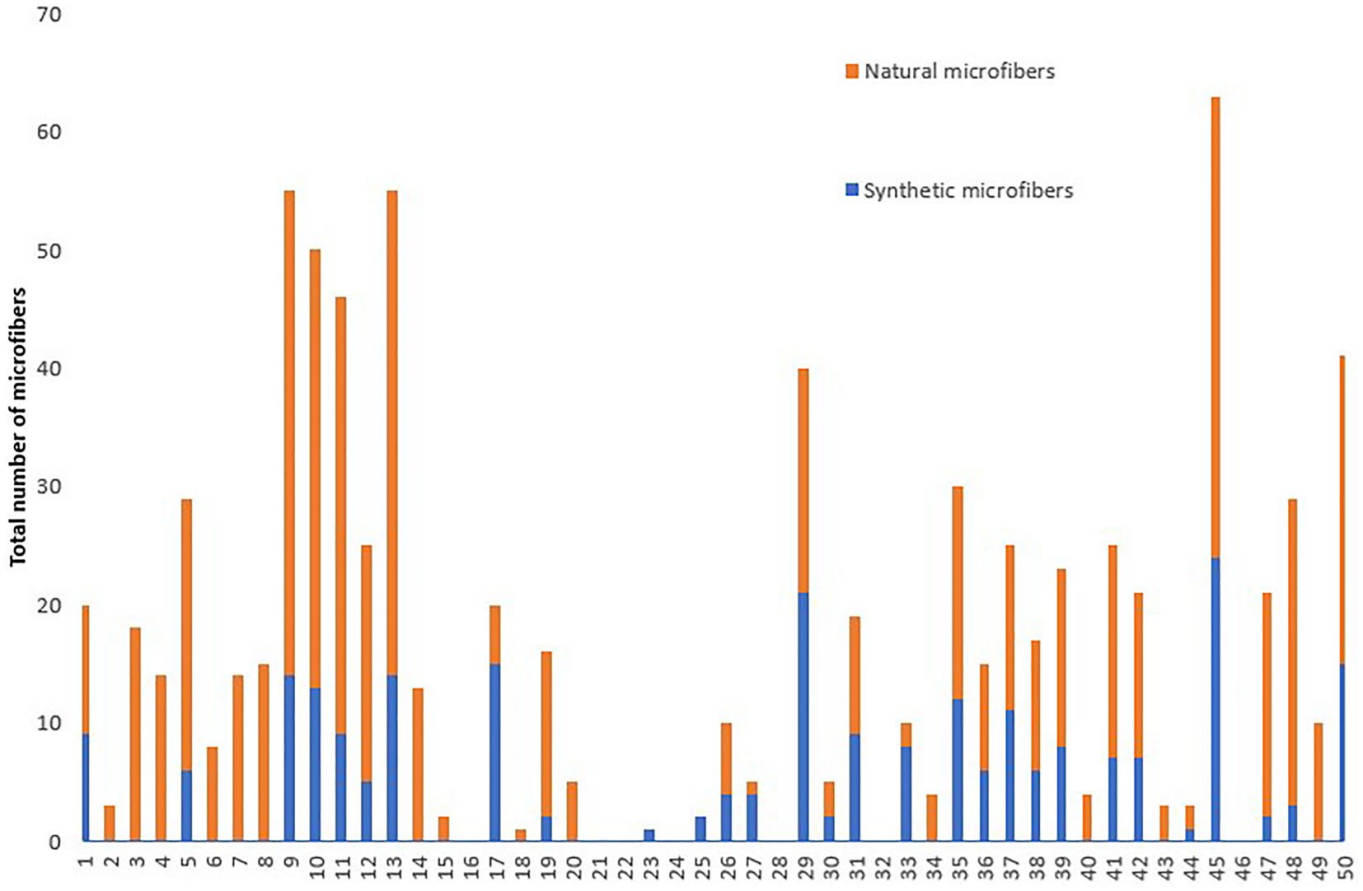
Discrimination into synthetic and naturals microfibers of the total number of microfibers found in analyzed mussels.

The adopted procedure allows to discriminate among natural and synthetic microfibers using their morphological features and to detect microfiber with a length in the range of 30–4900 µm. The minimum dimension limit is due to the possibility to detect the presence of typical morphological features of natural fibers along the fiber axis and/or to highlight the presence of defibrillation phenomena in degraded natural microfiber terminations. The procedure allows the visual determination of colored microfibers. Microfibers (both natural and synthetic) were present in 74% of mussel samples, ranging from 2 to 63 microfibers/individual and from 0.47 to 12.12 microfibers/g wet weight (w.w.) The mean number of microfibers observed was 14.57 microfibers/individual, corresponding to 3.13 microfibers/g w.w.. If considering only the samples containing microfibers, on average mussels showed 21.35 microfiber/individual, corresponding to 4.24 microfibers/g w.w. Natural microfibers, classified according to the evaluation of fiber morphologies, were the most numerous (58%) among the isolated microfibers (Fig. 1).

It was also found an aliquot of micro-debris, of about 18%, which could not be identified.

Chemical identification of microfibers and other micro-debris using vibrational spectroscopies such as FTIR and Raman spectroscopies was not directly applicable to the analysed samples. The employment of cellulose nitrate filters, useful for the filtration of this type of sample, interfered with signal detection. In fact, the used filters are not transparent and absorb IR radiation leading to absorption bands in the FTIR spectrum that cause strong spectral interference hindering spectra acquisition in both transmission, reflection, and ATR mode. Spectral interference, e.g. fluorescence, occurred also using Raman spectroscopy. The choice of these filters was due to the amount of filtered materials and to avoid filter clogging.

**Figure 2:**
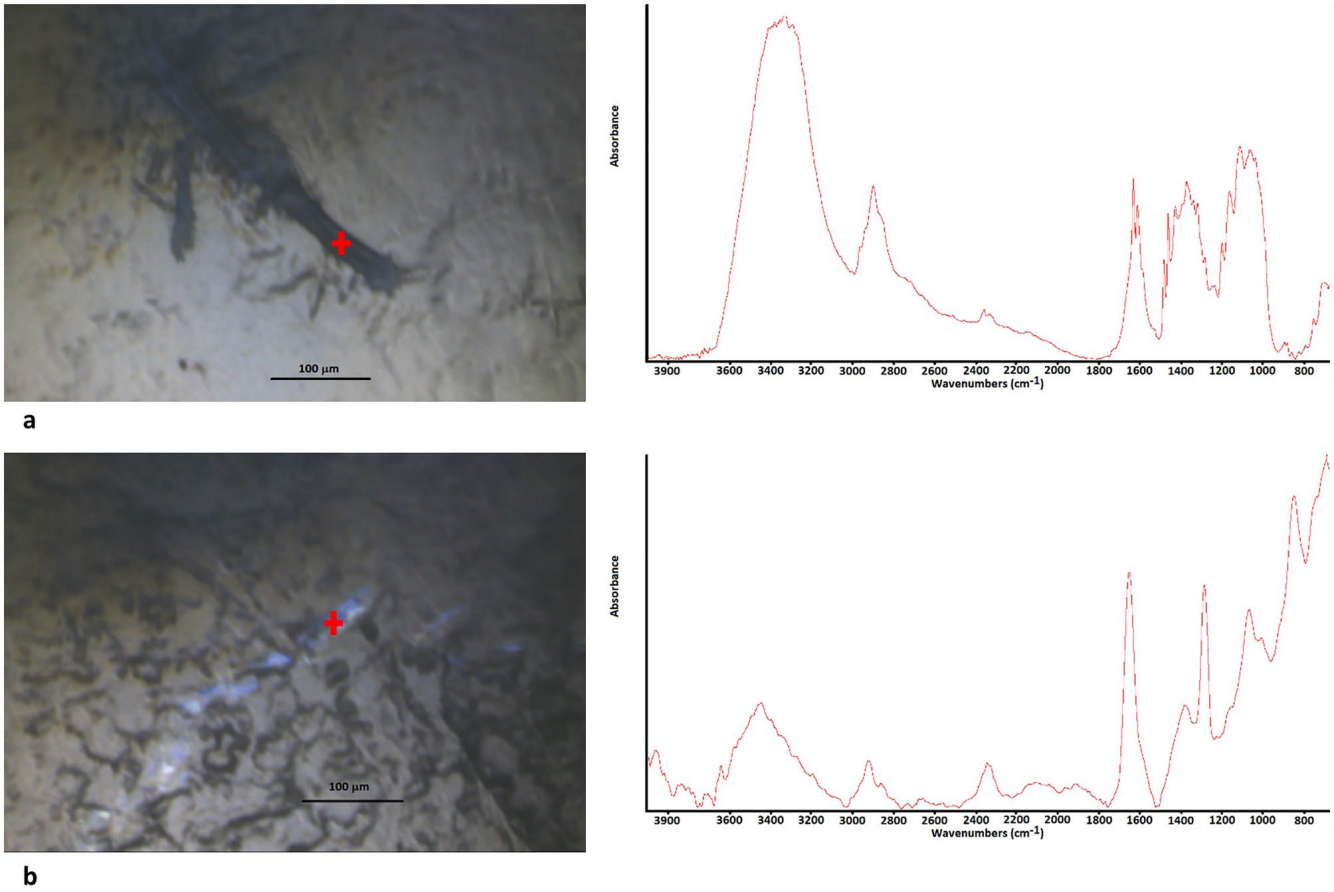
FTIR microspectroscopy of microfiber on KBr disc: (**a**) the microfiber with irregular shape and termination was of cellulosic nature; (**b**) the coloured microfibers with regular diameter was of polyamide nature.

The presence of spectral interference from the substrate, in addition to a so a large quantity of detected microfibers, compromised the ability to obtain compositional information of the microfibers. Therefore, to confirm the nature of microfibers previously classified according to their morphological characteristics, some microfibers present on the filter surface were deposited on KBr disc using ethanol. The FTIR analysis allows to confirm that the microfiber did not have uniform diameter, twisting and irregular termination is of natural origin (Fig. 2a), while microfiber with a smooth surface and cylindrical shape is synthetic (Fig. 2b). In fact, the FTIR spectrum reported in Fig. 2a is characterised by the presence of absorption bands in the range 3600–3000 cm^−1^, due to OH-stretching vibrations arising from hydrogen bonding in cellulose, bands at 2910 and 2850 cm^−1^, to CH_2_ asymmetrical and symmetrical stretching respectively, bands at, 1634 and 1427 cm^−1^ due to, amide I and CH_2_ scissoring, respectively. The 11,620, 1110, 10,607 and 1035 cm^−1^ absorption bands were attributed to anti-symmetrical bridge C–O–C stretching, anti-symmetric in-plane stretching band, C–O stretch. In the spectrum reported in Fig. 2b characteristic absorption bands of polyamide 66 are detectable. Bands due to NH stretching, CH_2_ stretching vibrations, C=O stretching, and combined NH scissoring and CN stretching vibrations were observed at 3450, at 29,250 and 2855 cm^−1^, 1650 and 1550 cm^−1^, respectively.

### Mussel size and microfiber content

Ingestion of microfibers by *M. galloprovincialis* varied within size classes (group I: < 4.5 g w.w.; group II: from 4.5 to 6.0 g w.w.; group III: > 6.0 g w.w.). When considering the microfiber number (both natural and synthetic) per g w.w., the smallest individuals (group I and II) had significantly higher microfiber levels (mean 6.22 items/g w.w.) compared to larger individuals (group III; 3.65 items/g w.w.). Statistical analysis, performed using post-hoc pairwise comparison between groups with non-parametric Kurskal–Wallis test (KW), pointed out a significant difference among the amount of microfiber per g w.w. detected during the microscopical analysis as a function of mussel size (g w.w.) (KW–group I-group III: *p* = 0.029; KW–group II-group III: *p* = 0.033; Table S1–S2), except for microfibers per g w.w. detected in specimens with the smallest size (KW–group I-group II: *p* = 0.962).

**Figure 3:**
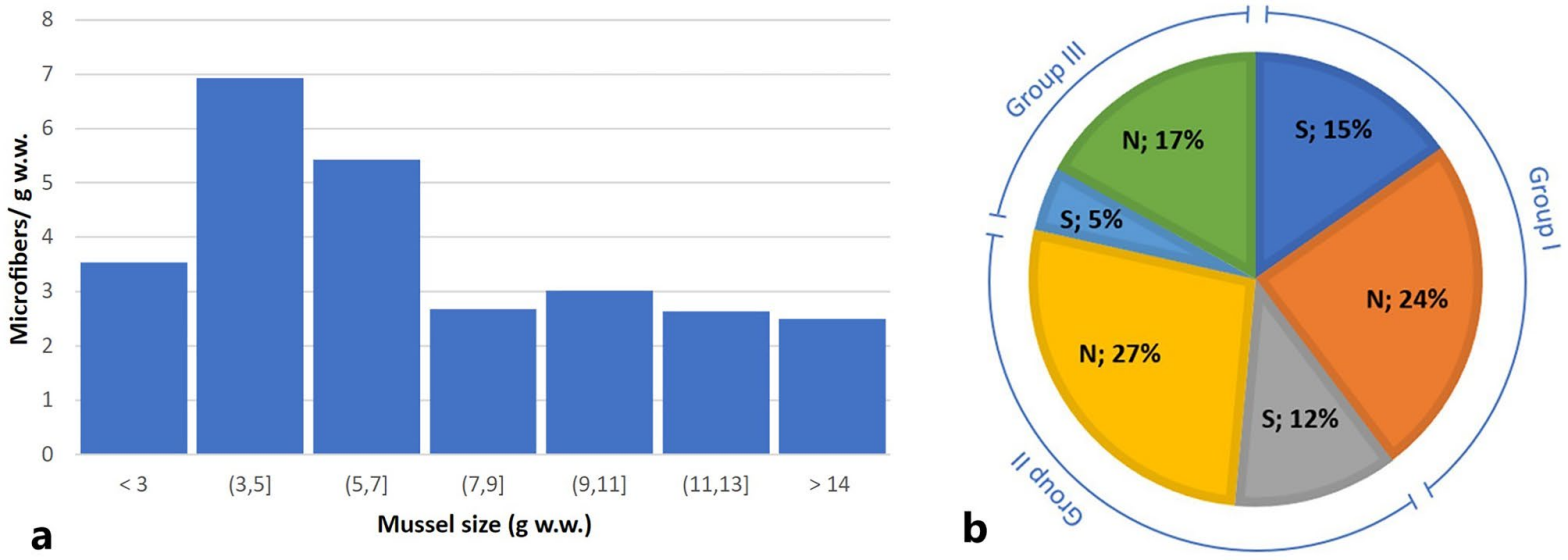
(**a**) Distribution of microfibers per g w.w. in different size mussels (**b**) Percentage distribution of natural (N) and synthetic (S) microfibers per g w.w. within size classes.

The negative correlation identified between mussel size and microfiber content (*r*(48) = − 0.339, *p* = 0.016, Table S3) (Fig. 3a) suggests that small mussels filter water more efficiently respect to larger specimens^14,34^. Microfibers distribution within the three size classes is reported in Fig. 3b.

A correlation test between microfiber type and mussel size was performed considering differences between natural and synthetic microfibers. A negative correlation was obtained between mussel size and synthetic microfibers/g w.w. (*r*(50) = 0.359, *p* = 0.010, Table S4) suggesting that larger specimens contain less number of synthetic microfibers. No significant correlation between mussel size and natural microfibers/g w.w. was found (*r*(50) = − 0.187, *p* = 0.194, Table S5).

Statistical analysis was performed to evaluate the difference between the sampling period of the mussels and the number of microfiber/g w.w. Mussels were divided into 4 groups according to the sampling period^39^ in group A: May–June, group B: August–September, group C: October, group D: February–March. No significant difference among the above reported groups and microfiber/individual was found (KW—p = 0549). Despite the statistical results a trend could be observed, in fact, microfibers/individual increases from group A to group C, but not significantly (r (37) = 0.207, p = 0.219 Table S6).

### Characteristics of recovered microfibers

**Figure 4:**
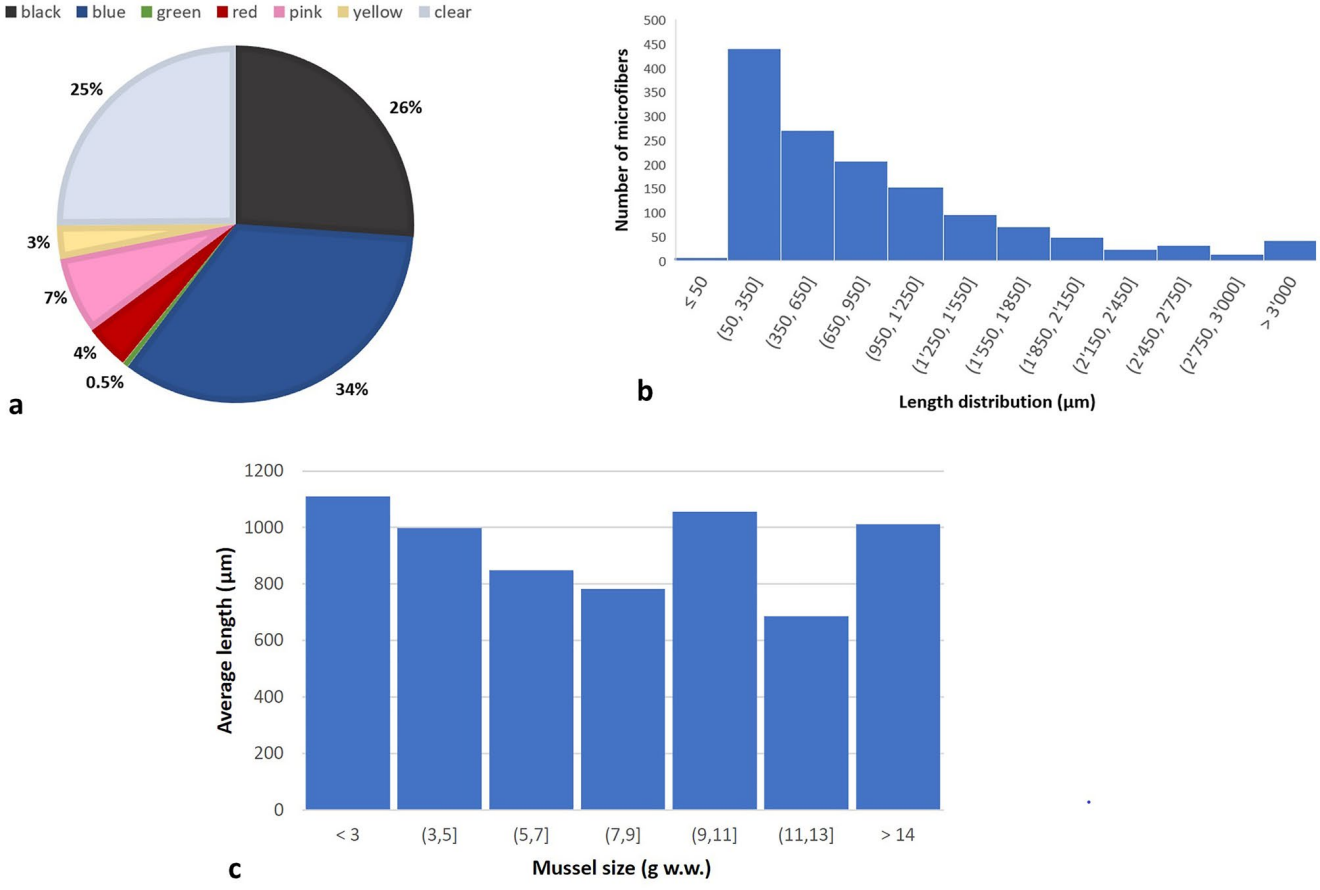
(**a**) Percentage distribution of microfibers colours in mussel samples; (**b**) Distribution of microfibers length recovered from the filters surface; (**c**) Plot of average microfiber length (µm) and mussel size (g w.w.).

The most common colours of microfibers (both natural and synthetic) were blue (34%), black (26%), and clear (25%), and a few were pink, red, yellow, and green. In Fig. 4a the colour distribution of the detected microfiber is reported.The average microfiber length was evaluated along with the standard deviation, through the measurements of the length of all the detected microfibres analysing optical micrographs by using Image J (release 1.43 u). The average lengths were determined to be 731.16 and 985.14 µm for synthetic and natural microfibers, respectively. Overall, 67% of the microfibers fell within the length range of 50 µm–1 mm, while a small percentage (33%) had a size between 1–5 mm (Fig. 4b). No significant difference was detected between the synthetic and natural microfiber lenght (KW—p = 0.479), as well as between the average microfibers length and mussel size groups (g w.w.) (ANOVA–F(2,47) = 2.242, *p* = 0.118; Table S7). Despite the statistical results (*r*(48) = − 0.159, *p* = 0.271; Table S8), negative correlation between microfiber length and mussel size could be observed (Fig. 4c).

## Discussion

In the present study, a versatile approach, based on the application of an optical method, was implemented, and applied to evaluate microfiber occurrence, size, colour, and nature. This approach allowed the monitoring of microfiber contamination in farmed mussels (*Mytilus galloprovincialis*) from the Tyrrhenian Sea on sale for human consumption.

Some studies have already reported the presence of microfibers in mussels collected over the world. In detail, mussels collected along the Belgian coast and purchased from department stores have been found to contain between 0.26 and 0.51 microfibers/g of tissue ranging from 200 µm up to 1500 µm size^40^, while greater quantities of microfibers (up to about 5 items/g, from 5 µm to 5 mm) have been detected in commercial bivalves from a fishery market in China^41^, and from 30 to 70 microfibers per individual have been found in *M. edulis* collected from a Nova Scotian harbour and purchased from an aquaculture site, respectively^17^. In the present study, the number of microfibers observed using the optical method in mussels from the Tyrrhenian Sea was lower than those reported by Avio et al.^13^ in *M. galloprovincialis* from the Central Adriatic Sea (20.8 ± 8.88 microfibers/individual), but generally higher than those reported by Klasios et al.^27^ in mussels from Canada.

Microfiber contamination (2594 microfibers per km^2^) in the western Mediterranean had already been reported^42^, but several factors can affect the levels of exposure in filter feeding organisms. Farmed mussels may contain more microfibers than wild mussels since they grow on polypropylene lines and are often cultured in coastal areas^9,17^. The depuration that farmed bivalves can undergo before the sale may help mussels to eliminate ~85% of filtered microplastic, but small microfibers (50 µm–1 mm) are little affected by depuration and are more accumulated by mussels than larger ones (1–5 mm)^8,43^. According to literature, mussels from the Tyrrhenian Sea have shown the occurrence of high levels (67%) of small microfibers (50 µm–1 mm), which not being retained by wastewater treatment plants are widespread along urbanized coasts^10,17^.

The prevalence in mussels of dark and clear colours also reflects the contamination of surrounding water, being blue, transparent, and black the most common colours of microfibers in seawater^16,18^. Mussels filter and retain particles regardless of their colour, and this could explain that the proportion of microfiber colours in mussels is similar to those found in the waters. On the contrary, fishes show more coloured microplastics in their gastrointestinal tracts because they mistakenly ingest debris resembling their natural prey^44^.A negative correlation was found between the levels of microfibers and the mussel weight^14^. Our results confirm this relationship which can be explained by the fact that in *Mytilus* species, pumping and filtration rates decrease with higher soft tissue mass^14^. Larger mussels may trap a lower number of fibers due to size dependent filtration rates; consequently, smaller mussels contain more microfibers than larger mussels^14,38^. The mussel’s metabolic requirements show a typical seasonal pattern. There is a spawning period in spring and summer, followed by a period of gonad development in autumn and winter^45^, during which weight loss and depletion of both protein and lipid contents are observed ^39,46^. However, in agreement with Ding et al.^47^, no significant difference between the mussel sampling period and microfiber /g w.w. was evaluated. Further studies are needed to better understand the physiological factors that determine the extent of microfiber exposure by marine biota and the ecological significance of the phenomenon^17,48^.

It should also be emphasized that many species of the *Mytilus* genus are of substantial commercial value as seafood items, and there are concerns about microplastic, including synthetic microfiber, transfer, and exposure in humans via ingestion^14^. Moreover, many studies are only focused on plastic microlitter and rarely include non-plastic debris^38^, which accounted for more than 50% of the total microfibers observed in the present study. Natural microfibers constitute a large portion of microfibers that may enter the marine environment via similar sources to the plastic microfibers^48,49^. Natural microfibers can carry harmful chemicals and release them even more readily than synthetic fibers due to faster degradation times^10^. However, far less is known about natural microfibers, despite the evidence they pose a risk to the environment^10,38^.

Researchers sometimes exclude both natural and synthetic microfibers from analysis due to background contamination^50–53^. This may hinder the evaluation of microfiber distribution in the marine environment and their implications on marine biota, because excluding microfibers from a study certainly reduced the number of microplastics reported in bivalve and fish digestive tracts^18,54^. Airborne microfibers contamination of field samples can occur at any time during collection, processing, and microscopic observation^14^. Thus, quality assurance measures should be more strictly applied when working with microfibers, and in addition to procedural blanks, also field blanks should be adopted in order to assess important sources of contamination during sampling procedures^55^.

Moreover, for a more comprehensive assessment a standardized protocol for the quantification and monitoring of microplastics, with a particular focus on synthetic microfibers, should be developed. The optical microscopy technique applied to assess the occurrence and the nature of natural or synthetic microfibers, considering their morphological features, would be a versatile approach to evaluate the contamination in commercial seafood, and a fast and easy detection method to gain information about the presence of fibrous microplastics in biological matrices. The most frequently employed methodologies to identify microfiber composition are based on spectroscopy measurements, such as FTIR and Raman spectroscopy that require sample preparation and appropriate substrates, to prevent interference of the surrounding environment, in addition to be expensive instrumentation^36^. In this respect, the adopted optical method was useful to quantify and discriminate microfibers of natural and synthetic origin in mussels. Fragmentation and physical changes on fiber surfaces may occur due to photodegradation, oxidation and mechanical abrasion^56,57^. There are very limited data on the rates at which different polymers degrade and fragment under varying environmental conditions^56–58^. After 14 days with UV exposure, both polyamide and polyester fibers showed signs of degradation with the formation of holes or pitting on their surfaces, while fibrils appeared on wool fiber surface^58^. Cotton fibers also could be broken down in their structural microfibrils during natural aging^59^. However, the degraded microfibers still present the morphological features to be exploited for identification purpose.

The results pave the way to the development of a fast, automated analytical tool to the identification of microfibers based on the usage of microfiber morphological features and artificial intelligence approaches using morphological features as input variables.

## Materials and methods

### Materials

Sodium Chloride, Hydrogen Peroxide solution 30%, and Potassium hydroxide were purchased from Carlo Erba (Val De Reuil, France). Cellulose nitrate (pore size 8 µm) and cellulose acetate (pore size 0.45 µm) filters were provided by Sartorius Stedim Biotech (Gottingen, Germany). The filtrating system was supplied by Advantec (Dublin, CA 94,568, USA).

### Sample collection

A number of 50 samples of farmed mussels (*M. galloprovincialis)* from the Tyrrhenian Sea (FAO subarea 37.1, division 37.1.3) were collected throughout 2020–2021 from fish markets located in Campania Region, Italy. Before selling, mussels underwent a depuration treatment according to EU Regulation 853/04.

Each sample was wrapped in alluminium foil and stored at − 20 °C until further processing. At the time of analyses, mussels were defrosted for ~ 30 min, and then washed with distilled water, previously filtered on 0.45 µm cellulose acetate membrane, to remove associated debris. The sample length was recorded with calibre, and the entire soft tissues of each shellfish were weighted after shell removal.

### Tissue digestion

Mussel samples were treated using an extraction method previously developed^60^. In detail, the soft tissues of each mussel were inserted into a glass Erlenmeyer flask (one mussel per flask) and submerged with a 10% KOH solution, approximately triple the volume of the tissue, and stored overnight in an oven at 45 °C. After the digestion, each sample was added to 250 ml NaCl prefiltered hypersaline solution (1.2 g/cm^3^), stirred, and decanted for 10 min. The overlying water was vacuum filtered using cellulose nitrate filters (pore size 8 μm). The filtration step was carried out twice in order to obtain a better extraction performance. The filters with retained materials were removed from the filtration device using clean stainless-steel tweezers and placed into clean Petri dishes. To adequately digest all tissue residues, a 15% H_2_O_2_ solution was added to the membranes and allowed to dry in oven (45 °C, overnight), before the microscopical observation.

One blank sample, which undertook all the steps of sampling analyses, was run for every batch of mussels processed.

### Filter observation and identification of microfibers

After the digestion and filtering process, an optical method was used to identify and count the number of microfibers. Filters were analysed using a LEICA M205C light microscope, with a magnification of 0.78–16x  to evaluate the presence of microfibers and to discriminate among natural and synthetic microfibers using their morphological features^61,62^.

Further microfibers were classified by colour and length. For comparison, standard uncolored microfibers of natural and synthetic origin such as wool, cotton, flax, polyamide and polyester were analysed using a LEICA M205C light microscope. These micrographs were used as references to highlight the microfiber surface features to be used during identification of the analysed samples, according to Rodríguez-Romeu et al.^63^. The typical features exhibited by natural and synthetic standard microfibers during light microscopy observation are summarized in Table 1.

**Table 1:** Typical morphological features of natural and synthetic microfibers.

Microfiber type	Micrograph	Morphological feature
Wool		Scale patterns
Cotton		Flat, ribbon-like, with irregular twisting around the fiber axis (convolutions)
Flax		Cross markings and dislocation, very narrow lumen
Polyester		Fine, regular longitudinal structure, circular cross-section
Polyamide		Fine, regular longitudinal structure and featureless, circular cross-section

**Figure 5:**
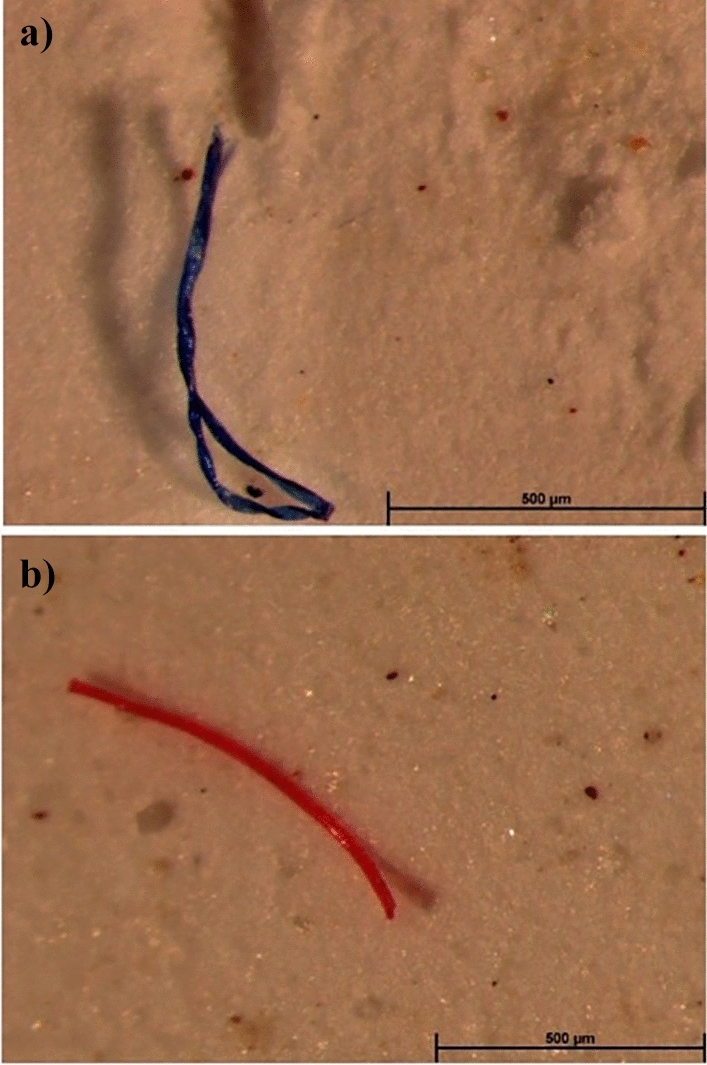
Optical micrographs at different magnification of two microfibers detected on filter surface: (**a**) twisting and convolutions features of a natural microfiber and (**b**) cylindrical shape and smooth surface of a synthetic microfiber.

For sample analysis and micrographs acquisition the following procedure was employed: (1) each filter was considered divided in four frames, as described in Supplementary Material – Fig. S1; (2) each frame was captured with a magnification of 0.78x (Fig. S2a); (3) different magnifications were used in order to capture details (Fig. S2b). The typical morphological features of the textile fibers were used to classify the detected microfibers as synthetic or natural ones. There are major differences between natural and synthetic fibers, such as the morphology of natural fibers is more complex than synthetic fibres^64^. Microfibers that did not show a uniform diameter while twisted upon themselves like flat ribbons were classified as natural fibers (Fig. 5a). Meanwhile, microfibers with a smooth and shiny surface that showed a cylindrical shape were identified like synthetic fibers (Fig. 5b).

Approximately 30 min were spent for the examination of each filter. The quantification of detected microfibers in mussel samples was corrected by subtracting the number of microfibers determined in the blank samples.

To confirm the proposed approach some microfibers were characterized using Thermo Scientific iN10 FTIR Microscope. Due to the filter nature the FTIR analysis of microfiber on the filter surface was not allowed thus, before FTIR analysis, microfibers on the filter surface were re-dispersed in ethanol and the dispersion was dropped on kBr disc.

### Quality control and assurance

As contamination precaution, cotton lab coats were worn when handling samples. All of the liquids and solutions were filtered over cellulose acetate filter (pore size 0.45 μm) before use, and all work surfaces and apparatus were cleaned with filtered water. Glass containers and beakers were rinsed three times with filtered water. The samples were covered with aluminium foil during digestion, stirring, decantation and filtration steps. During the microscopical observation, cotton lab coats and nitrile gloves were worn. Two control dishes with kBr discs were placed near the sample during the FTIR analyses in order to collect any atmospheric or handling-related contamination that may occur. No contamination was detected.

### Statistical analysis

Statistical analysis of the number and length of microfibers observed in the samples was carried out by using IBM®SPSS® Statistics software. The data were tested for normality using Shapiro–Wilk test and for homogeneity of variance using Levene’s test. One-way Analysis of Variance (ANOVA) was performed to assess significant differences among the data. When data did not comply with the assumption of normality, a post-hoc pairwise comparison between the groups with non-parametric KW test was performed. To determine the relationship between the mussel size and the values of number and length of microfibers observed, Pearson correlation test was performed. A 5% significance level was considered for all the statistical tests (p values < 0.05 indicate significant differences among the data).

## Supplementary Information

Supplementary Information.

## Data Availability

The datasets used and/or analyzed during the current study available from the corresponding author on reasonable request.

## References

[1] Azevedo-Santos VM, Gonçalves GRL, Manoel PS, Andrade MC, Lima FP, Pelicice FM (2019). Plastic ingestion by fish: A global assessment. Environ. Pollut..

[2] Avio CG, Cardelli LR, Gorbi S, Pellegrini D, Regoli F (2017). Microplastics pollution after the removal of the Costa Concordia wreck: First evidences from a biomonitoring case study. Environ. Pollut..

[3] Cole M, Liddle C, Consolandi G, Drago C, Hird C, Lindeque PK, Galloway TS (2020). Microplastics, microfibres and nanoplastics cause variable sub-lethal responses in mussels (Mytilus spp.). Mar. Pollut. Bull..

[4] Digka N, Tsangaris C, Torre M, Anastasopoulou A, Zeri C (2018). Microplastics in mussels and fish from the Northern Ionian Sea. Mar. Pollut. Bull..

[5] Lusher A, Welden N, Sobral P, Cole M (2017). Sampling, isolating and identifying microplastics ingested by fish and invertebrates. Anal. Methods.

[6] Pittura L, Avio CG, Giuliani ME, d’Errico G, Keiter SH, Cormier B, Gorbi S, Regoli F (2018). Microplastics as vehicles of environmental PAHs to marine organisms: Combined chemical and physical hazards to the mediterranean mussels, *Mytilus galloprovincialis*. Front. Mar. Sci.

[7] Santillo D, Miller K, Johnston P (2017). Microplastics as contaminants in commercially important seafood species: Microplastics in seafood. Integr. Environ. Assess Manag..

[8] Kolandhasamy P, Su L, Li J, Qu X, Jabeen K, Shi H (2018). Adherence of microplastics to soft tissue of mussels: A novel way to uptake microplastics beyond ingestion. Sci. Total Environ..

[9] Li J, Qu X, Su L, Zhang W, Yang D, Kolandhasamy P, Li D, Shi H (2016). Microplastics in mussels along the coastal waters of China. Environ. Pollut..

[10] Acharya S, Rumi SS, Hu Y, Abidi N (2021). Microfibers from synthetic textiles as a major source of microplastics in the environment: A review. Text. Res. J..

[11] De Falco F, Gullo MP, Gentile G, Di Pace E, Cocca M, Gelabert L, Brouta-Agnésa M, Rovira A, Escudero R, Villalba R, Mossotti R, Montarsolo A, Gavignano S, Tonin C, Avella M (2018). Evaluation of microplastic release caused by textile washing processes of synthetic fabrics. Environ. Pollut..

[12] Volgare M, De Falco F, Avolio R, Castaldo R, Errico ME, Gentile G, Ambrogi V, Cocca M (2021). Washing load influences the microplastic release from polyester fabrics by affecting wettability and mechanical stress. Scientific Reports.

[13] Avio CG, Pittura L, d’Errico G, Abel S, Amorello S, Marino G, Gorbi S, Regoli F (2020). Distribution and characterization of microplastic particles and textile microfibers in Adriatic food webs: General insights for biomonitoring strategies. Environ. Pollut..

[14] Catarino AI, Macchia V, Sanderson WG, Thompson RC, Henry TB (2018). Low levels of microplastics (MP) in wild mussels indicate that MP ingestion by humans is minimal compared to exposure via household fibres fallout during a meal. Environ. Pollut..

[15] Jemec A, Horvat P, Kunej U, Bele M, Kržan A (2016). Uptake and effects of microplastic textile fibers on freshwater crustacean Daphnia magna. Environ. Pollut..

[16] Mankin C, Huvard A (2020). Microfibers in Mytilus species (Mollusca, Bivalvia) from Southern California harbors, beaches and supermarkets. Am. J. Undergrad. Res..

[17] Mathalon A, Hill P (2014). Microplastic fibers in the intertidal ecosystem surrounding Halifax Harbor Nova Scotia. Mar. Pollut. Bull..

[18] Gago J, Carretero O, Filgueiras AV, Viñas L (2018). Synthetic microfibers in the marine environment: A review on their occurrence in seawater and sediments. Mar. Pollut. Bull..

[19] Mateos-Cárdenas A, O’Halloran J, van Pelt FNAM, Jansen MAK (2021). Beyond plastic microbeads—Short-term feeding of cellulose and polyester microfibers to the freshwater amphipod *Gammarus duebeni*. Sci. Total Environ..

[20] Choi JS, Kim K, Hong SH, Park K. Il, Park JW (2021). Impact of polyethylene terephthalate microfiber length on cellular responses in the Mediterranean mussel *Mytilus galloprovincialis*. Mar. Environ. Res..

[21] Baroja E, Christoforou E, Lindström J, Spatharis S (2021). Effects of microplastics on bivalves: Are experimental settings reflecting conditions in the field?. Mar. Pollut. Bull..

[22] Christoforou E, Dominoni DM, Lindström J, Stilo G, Spatharis S (2020). Effects of long-term exposure to microfibers on ecosystem services provided by coastal mussels. Environ. Pollut..

[23] Mercogliano R, Avio CG, Regoli F, Anastasio A, Colavita G, Santonicola S (2020). Occurrence of microplastics in commercial seafood under the perspective of the human food chain. A review. J. Agric. Food Chem..

[24] Renzi M, Guerranti C, Blašković A (2018). Microplastic contents from maricultured and natural mussels. Mar. Pollut. Bull..

[25] Leslie, H. A., van Velzen, M. J. M. & Vethaak, A. D. Microplastic survey of the Dutch environment: Novel data set of microplastics in North Sea sediments, treated wastewater effluents and marine biota. Netherlands 1–30 (2013).

[26] Berglund E, Fogelberg V, Nilsson PA, Hollander J (2019). Microplastics in a freshwater mussel (*Anodonta anatina*) in Northern Europe. Sci. Total Environ..

[27] Klasios N, De Frond H, Miller E, Sedlak M, Rochman CM (2021). Microplastics and other anthropogenic particles are prevalent in mussels from San Francisco Bay and show no correlation with PAHs. Environ. Pollut..

[28] Pennino MG, Bachiller E, Lloret-Lloret E, Albo-Puigserver M, Esteban A, Jadaud A, Bellido JM, Coll M (2020). Ingestion of microplastics and occurrence of parasite association in Mediterranean anchovy and sardine. Mar. Pollut. Bull..

[29] Capone A, Petrillo M, Misic C (2020). Ingestion and elimination of anthropogenic fibres and microplastic fragments by the European anchovy (*Engraulis encrasicolus*) of the NW Mediterranean Sea. Mar. Biol..

[30] Caldwell J, Petri-Fink A, Rothen-Rutishauser B, Lehner R (2019). Assessing meso- and microplastic pollution in the Ligurian and Tyrrhenian Seas. Mar. Pollut. Bull..

[31] Palazzo L, Coppa S, Camedda A, Cocca M, De Falco F, Vianello A, Massaro G, de Lucia GA (2021). A novel approach based on multiple fish species and water column compartments in assessing vertical microlitter distribution and composition. Environ. Pollut..

[32] Bottari T, Savoca S, Mancuso M, Capillo G, GiuseppePanarello G, MartinaBonsignore M, Crupi R, Sanfilippo M, D’Urso L, Compagnini G, Neri F, Romeo T, Luna GM, Spanò N, Fazio E (2019). Plastics occurrence in the gastrointestinal tract of Zeus faber and *Lepidopus caudatus* from the Tyrrhenian Sea. Mar. Pollut. Bull..

[33] Savoca S, Capillo G, Mancuso M, Bottari T, Crupi R, Branca C, Romano V, Faggio C, D’Angelo G, Spanò N (2019). Microplastics occurrence in the Tyrrhenian waters and in the gastrointestinal tract of two congener species of seabreams. Environ. Toxicol. Pharmacol..

[34] Savoca S, Bottari T, Fazio E, Bonsignore M, Mancuso M, Luna GM, Romeo T, D’Urso L, Capillo G, Panarello G, Greco S, Compagnini G, Lanteri G, Crupi R, Neri F, Spanò N (2020). Plastics occurrence in juveniles of *Engraulis encrasicolus* and *Sardina pilchardus* in the Southern Tyrrhenian Sea. Sci. Total Environ..

[35] Capillo G, Savoca S, Panarello G, Mancuso M, Branca C, Romano V, Spanò N (2020). Quali-quantitative analysis of plastics and synthetic microfibers found in demersal species from Southern Tyrrhenian Sea (Central Mediterranean). Mar. Pollut. Bull..

[36] Xu JL, Thomas KV, Luo Z, Gowen AA (2019). FTIR and Raman imaging for microplastics analysis: State of the art, challenges and prospects. TrAC Trend Anal. Chem..

[37] Athey SM, Erdle LM (2021). Are we underestimating anthropogenic microfiber pollution? A critical review of occurrence, methods, and reporting. Environ. Toxicol. Chem..

[38] Doucet CV, Labaj AL, Kurek J (2021). Microfiber content in freshwater mussels from rural tributaries of the Saint John River, Canada. Water Air Soil Pollut..

[39] Fernández A, Grienke U, Soler-Vila A, Guihéneuf F, Stengel DB, Tasdemir D (2015). Seasonal and geographical variations in the biochemical composition of the blue mussel (*Mytilus edulis* L) from Ireland. Food Chem..

[40] De Witte B, Devriese L, Bekaert K, Hoffman S, Vandermeersch G, Cooreman K, Robbens J (2014). Quality assessment of the blue mussel (*Mytilus edulis*): Comparison between commercial and wild types. Mar. Pollut. Bull..

[41] Li J, Yang D, Li L, Jabeen K, Shi H (2015). Microplastics in commercial bivalves from China. Environ. Pollut..

[42] Faure F, Saini C, Potter G, Galgani F, de Alencastro LF, Hagmann P (2015). An evaluation of surface micro- and mesoplastic pollution in pelagic ecosystems of the Western Mediterranean Sea. Environ. Sci. Pollut. Res..

[43] Fernández B, Albentosa M (2019). Insights into the uptake, elimination and accumulation of microplastics in mussel. Environ. Pollut..

[44] Ríos MF, Hernández-Moresino RD, Galván DE (2020). Assessing urban microplastic pollution in a benthic habitat of Patagonia Argentina. Mar. Pollut. Bull..

[45] Smaal AC, Vonck APMA (1997). Seasonal variation in C, N and P budgets and tissue composition of the mussel *Mytilus edulis*. Mar. Ecol. Prog. Ser.

[46] Okumuş İ, Stirling HP (1998). Seasonal variations in the meat weight, condition index and biochemical composition of mussels (*Mytilus edulis* L.) in suspended culture in two Scottish sea lochs. Aquaculture.

[47] Ding J, Sun C, He C, Li J, Ju P, Li F (2021). Microplastics in four bivalve species and basis for using bivalves as bioindicators of microplastic pollution. Sci. Total Environ..

[48] Halstead JE, Smith JA, Carter EA, Lay PA, Johnston EL (2018). Assessment tools for microplastics and natural fibres ingested by fish in an urbanised estuary. Environ. Pollut..

[49] Yu X, Ladewig S, Bao S, Toline CA, Whitmire S, Chow AT (2018). Occurrence and distribution of microplastics at selected coastal sites along the southeastern United States. Sci. Total Environ..

[50] Besseling E, Foekema EM, Van Franeker JA, Leopold MF, Kühn S, Bravo Rebolledo EL, Heße E, Mielke L, IJzer J, Kamminga P, Koelmans AA (2015). Microplastic in a macro filter feeder: Humpback whale Megaptera novaeangliae. Mar. Pollut. Bull..

[51] Bosshart S, Erni-Cassola G, Burkhardt-Holm P (2020). Independence of microplastic ingestion from environmental load in the round goby (*Neogobius*
*melanostomus*) from the Rhine River using high quality standards. Environ. Pollut..

[52] Compa M, Alomar C, Mourre B, March D, Tintoré J, Deudero S (2020). Nearshore spatio-temporal sea surface trawls of plastic debris in the Balearic Islands. Mar. Environ. Res..

[53] Athey SN, Erdle LM (2022). Are we underestimating anthropogenic microfiber pollution? A critical review of occurrence, methods, and reporting. Environ. Toxicol. Chem..

[54] Covernton GA, Davies HL, Cox KD, El-Sabaawi R, Juanes F, Dudas SE, Dower JF (2021). A Bayesian analysis of the factors determining microplastics ingestion in fishes. J. Hazard. Mater..

[55] Prata JC, Castro JL, da Costa JP, Duarte AC, Rocha-Santos T, Cerqueira M (2020). The importance of contamination control in airborne fibers and microplastic sampling: Experiences from indoor and outdoor air sampling in Aveiro Portugal. Mar. Pollut. Bull..

[56] Andrady AL, Bergmann M, Gutow L, Klages M (2015). Persistence of plastic litter in the oceans. Marine Anthropogenic Litter.

[57] Booth, A. M., Kubowicz, S., Beegle-Krause, C., Skancke, J., Nordam, T., Landsem, E. & Jahren, S. Microplastic in global and Norwegian marine environments: Distributions, degradation mechanisms and transport. Miljødirektoratet M-918, 1–147 (2017).

[58] Sørensen L, Groven AS, Hovsbakken IA, Del Puerto O, Krause DF, Sarno A, Booth AM (2021). UV degradation of natural and synthetic microfibers causes fragmentation and release of polymer degradation products and chemical additives. Sci. Total Environ..

[59] Herrera LK, Justo A, Duran A, de Haro MC, Franquelo ML, Perez Rodríguez JL (2010). Identification of cellulose fibres belonging to Spanish cultural heritage using synchrotron high resolution X-ray diffraction. Appl. Phys. A.

[60] Mercogliano R, Santonicola S, Raimo G, Gasperi M, Colavita G (2021). Extraction and identification of microplastics from mussels: Method development and preliminary results. Ital. J. Food Saf..

[61] Allievo T (1908). Le Fibre Tessili di Applicazione Industriale.

[62] Hidalgo-Ruz V, Gutow L, Thompson RC, Thiel M (2012). Microplastics in the marine environment: A review of the methods used for identification and quantification. Environ. Sci. Technol..

[63] Rodríguez-Romeu O, Constenla M, Carrassón M, Campoy-Quiles M, Soler-Membrives A (2020). Are anthropogenic fibres a real problem for red mullets (*Mullus barbatus*) from the NW Mediterranean?. Sci. Total Environ..

[64] Ahmed F, Ibrahim Md, Mondal H, Ibrahim Md, Mondal H (2021). Introduction to natural fibres and textiles. The Textile Institute Book Series, Fundamentals of Natural Fibres and Textiles.

