# Supplementary material for: A versatile approach to evaluate the occurrence of microfibers in mussels Mytilus galloprovincialis
Source: Sci Rep. 2022 Dec 17;12:21827. doi: 10.1038/s41598-022-25631-2 (PMC9759576; doi:10.1038/s41598-022-25631-2)
Supplement: Supplementary file 1 — Supplementary Information. [file 41598_2022_25631_MOESM1_ESM.pdf]

A versatile approach to evaluate the occurrence of microfibers in mussels *Mytilus galloprovincialis*

Michela Volgare[1]†, Serena Santonicola[1,2]†, Mariacristina Cocca[1]*, Roberto Avolio[1], Rachele Castaldo[1], Maria Emanuela Errico[1], Gennaro Gentile[1], Gennaro Raimo[2], Maurizio Gasperi[2], Giampaolo Colavita[2]

[1] Institute of Polymers, Composites and Biomaterials, National Research Council of Italy, Via Campi Flegrei 34, 80078, Pozzuoli (NA) Italy

[2] Department of Medicine and Health Sciences "V. Tiberio", University of Molise, Via F. De Santis, 86100 Campobasso, Italy

* Corresponding author: Mariacristina Cocca (mariacristina.cocca@ipcb.cnr.it)

†These authors contributed equally to this work.

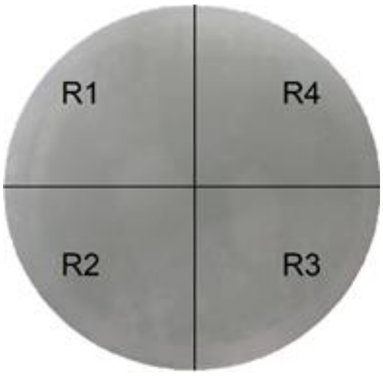

Fig. S1. Representation of filter surface divided in four ideal frames, related to "Filter observation and identification of microfibers" section.

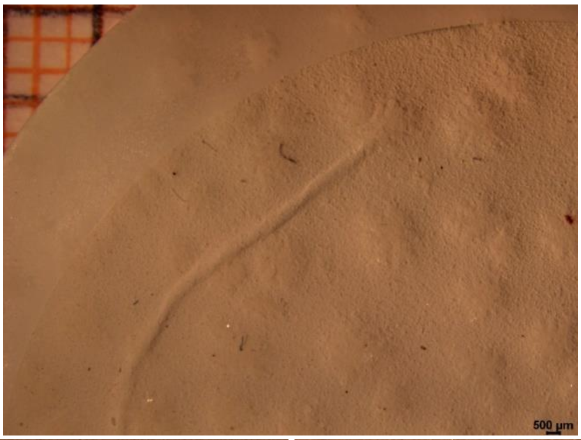

a)

b)

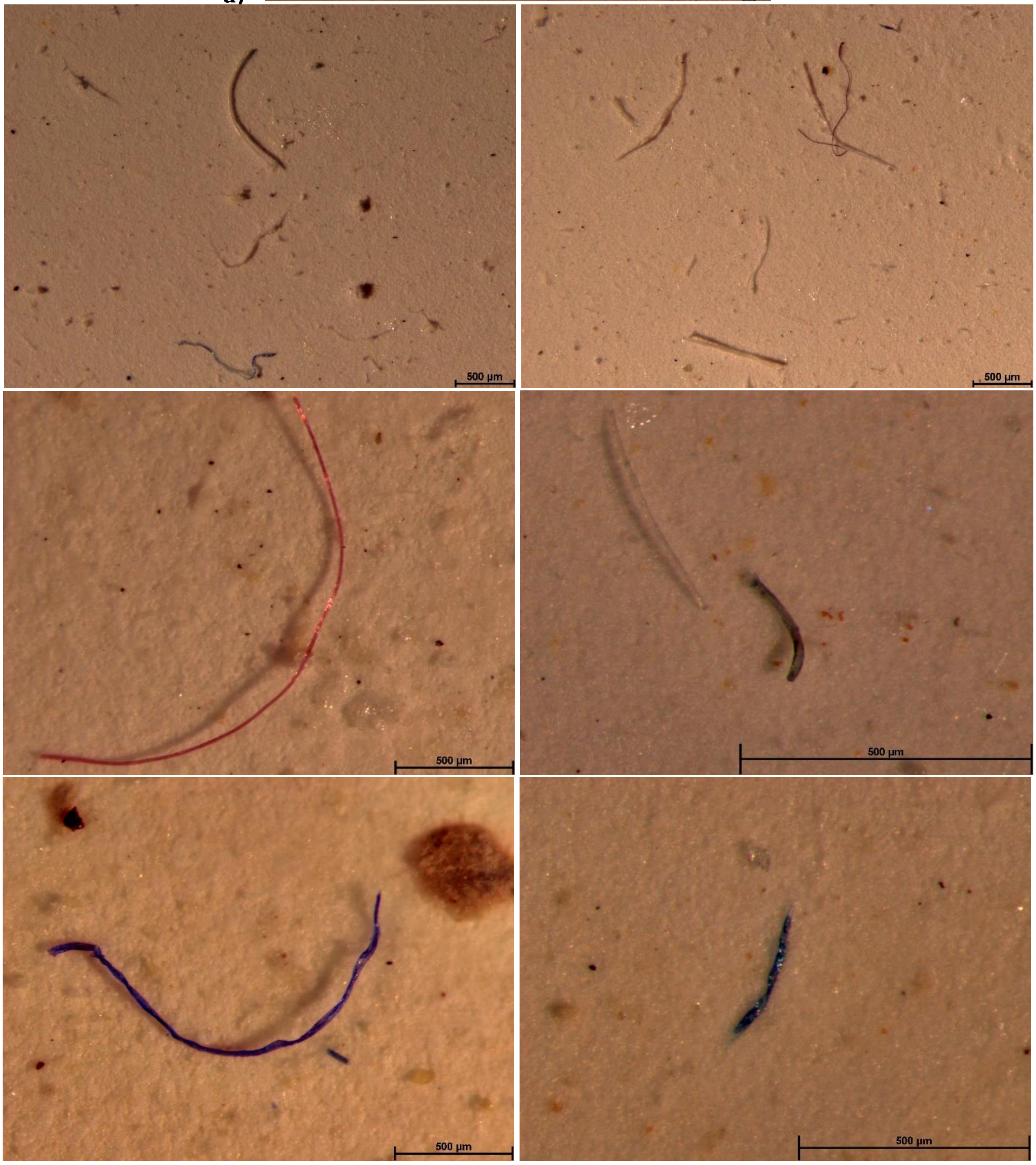

Fig. S2. a) Micrograph of filter surface with a magnification of 0.78x **b)** Micrographs of natural and synthetic fibers recovered from filter surface at different magnification, related to "Filter observation and identification of microfibers" section.

Standardization of the procedure

The whole procedure to analyse filter was following detailed: 1) observation of the filter surface using the minimum magnification to capture about ¼ of the filter area, which was selected as shown in Figure 1S; 2) within a quarter of the filter area, larger magnifications were carried out by moving the filter across the selected frame. The same procedure was carried out for all the frames selected and repeated for all samples observed. To standardize the used procedure, the same samples were observed several times, on different days, ascertaining that the fibers counted by the operator did not differ substantially.

Table S1. Kruskal-Wallis test on number of microfibers observed per g wet weight (MF/g w.w.) in mussels with size <4.5g w.w. (group I), from 4.5 to 6.0 g w.w. (group II), and >6.0g w.w. (group III), related to "Mussel size and microfiber content" section.

Hypothesis Test Summary

	Null Hypothesis	Test	Sig.	Decision
1	The distribution of MF/g w.w. is the same across categories of size.	Independent-Samples Kruskal-Wallis Test	.047	Reject the null hypothesis.

Asymptotic significances are displayed. The significance level is .050.

Table S2. Pairwise comparison of the Kruskal Wallis test on microfiber per gram wet weight (MF/g w.w.) observed in mussels with size <4.5g w.w. (group I), from 4.5 to 6.0 g w.w. (group II), and >6.0g w.w. (group III), related to "Mussel size and microfiber content" section.

Pairwise Comparisons of size

Sample 1-Sample 2	Test Statistic	Std. Error	Std. Test Statistic	Sig.	Adj. Sig.
III-II	10.820	5.078	2.131	.033	.099
III-I	11.055	5.078	2.177	.029	.088
II-I	.235	5.000	.047	.962	1.000

Each row tests the null hypothesis that the Sample 1 and Sample 2 distributions are the same.
Asymptotic significances (2-sided tests) are displayed. The significance level is .05.

Table S3. Pearson correlation among microfiber per gram wet weight (MF/g w.w.) and mussel size (g w.w.).

Correlations

		MF/g	size
MF/g w.w.	Pearson Correlation	1	-.339*
	Sig. (2-tailed)		.016
	N	50	50
size	Pearson Correlation	-.339*	1
	Sig. (2-tailed)	.016	
	N	50	50

*. Correlation is significant at the 0.05 level (2-tailed).

Table S4. Pearson correlation among mussel size (g w.w.) and synthetic microfibers per gram wet weight (MF/g w.w.)

Correlations

		Size	MF/g_Syn
Size	Pearson Correlation	1	-,359*
	Sig. (2-tailed)		,010
	N	50	50
MF/g_Syn	Pearson Correlation	-,359*	1
	Sig. (2-tailed)	,010	
	N	50	50

*. Correlation is significant at the 0.05 level (2-tailed).

Table S5. Pearson correlation among mussel size (g w.w.) and natural microfibers per gram wet weight (MF/g w.w.)

Correlations

		Size	MF/g_Nat
Size	Pearson Correlation	1	-,187
	Sig. (2-tailed)		,194
	N	50	50
MF/g_Nat	Pearson Correlation	-,187	1
	Sig. (2-tailed)	,194	
	N	50	50

Table S6. Pearson Correlation between number of microfibers found in samples and months in which samples themselves were recovered.

Correlations

		Months	Number
Months	Pearson Correlation	1	,207
	Sig. (2-tailed)		,219
	N	37	37
Number	Pearson Correlation	,207	1
	Sig. (2-tailed)	,219	
	N	37	37

Table S7. ANOVA on average length of microfibers recovered from filter after the digestion and filtering process performed on mussels with a size <4.5g w.w. (group I), from 4.5 to 6.0 g w.w. (group II), and >6.0g w.w. (group III), related to "Characteristics of recovered microfibers" section.

ANOVA

	Sum of Squares	df	Mean Square	F	Sig.
Between Groups	425833.311	2	212916.656	2.242	.118
Within Groups	4464369.353	47	94986.582		
Total	4890202.665	49			

Table S8. Pearson correlations among lengths of microfibers recovered from filter and mussel size (g w.w.).

Correlations

		Size	Length
Size	Pearson Correlation	1	-.159
	Sig. (2-tailed)		.271
	N	50	50
Length	Pearson Correlation	-.159	1
	Sig. (2-tailed)	.271	
	N	50	50
